# Suggestions Educational

**Published:** 1881-07

**Authors:** Ben. H. Pratt


### THE BISTOURY.

Vol. XVII. ELMIRA, N. Y., JULY, 1881. No. 2.

### Contributions.

### SUGGESTIONS EDUCATIONAL.

BEN. H. PRATT.

School Boards, as School Boards go, have no business to conduct the purely educational affairs of the districts which they represent. School Boards know as much about the sort of teachers that should be employed, the kind of instruction the young need, the value of the text books used, and the comparative merits of the various methods of teaching now in vogue, as a lawyer knows about what should be preached, as a preacher knows about conducting a case in court, as a civil engineer knows about treating a sick patient, or as a shoemaker knows about editing a newspaper. All that the average School Board is good for, is to manage the business of the district; and this will be enough for it to do if it does it well. School boards will do well enough as providers of the necessary buildings in which to conduct the schools, and should provide the means of paying for them, and for the instruction imparted within them, but beyond these the School Board's jurisdiction, if it do not great harm, prevents the accomplishment of much good. School directors are not generally, in fact seldom are, chosen because of their experience in teaching, or for their literary attainments. Nor should they be, if they are to manage the business of the schools. It has been very thoroughly demonstrated that our educators and our literary men do not make good business managers. In casting about for a good school director, the community does not look for a book-worm, nor for a blue-stocking. It looks for men with good, square business sense, downright honesty of purpose, and unquestionable interest in the welfare of the community. It doesn't care so much about what a man knows, as about what sort of a man he is. Nobody asks whether he talks good grammar, or whether he's quick at figures, or writes a good hand, or is a brilliant speaker or essayist. He must be one interested in the cause of public education, but need not be an educator in the school sense of the term. In fact, these booky and cultured fellows are almost invariably skipped when a school director is being looked for. The public has a sight more gumption than it often gets credit for. It doesn't look for business among the cultured any more than it looks for special intellectual culture among business men People are, take the average, practical, and understand that the chief thing required of a school director is management. It takes money to run schools, and it takes more than any community wants to pay to support them even under good business management; but if we trust our buildings and our schemes for paying for them to these theoretical fellows who are ever basking in the sunshine of Utopia, we'd soon have nothing to do with. We do choose good school directors, as a general thing, but the great mistake we make is in requiring them to superintend the literary and instructorial department as well as the business and managerial. Our public schools will never become what they ought to be until we divorce the teaching and the government from the business and the finance thereof. I think the above point is pretty clearly recognized by the general public. Almost any miscellaneous gathering of citizens—and such gathering is a very fair sample of the governing public—will admit that our School Boards ought to be made up of practical business men rather than of school men or university men, for although it has been demonstrated again and again that very fair schools may be and are continually conducted by men who have never had any special literary culture, yet it has been as frequently demonstrated that men of a purely intellectual and classical sort cannot sustain the most ordinary common schools if compelled to rely upon themselves and their own theories.

What I want to get at is this, that the business management only of our public school system should be put into the hands of our School Boards, and this should by law be made the extent of their jurisdiction. They should control the property of the district, and determine the best methods for acquiring, holding and improving that property. Theirs should be a purely business work, utterly divorced from that of teaching or selecting teachers, of selecting text books and mapping out courses of instruction, of governing and disciplining teachers and pupils, or doing anything save the managing of the business of the schools. For the literary and moral training of those whom the public places in the hands of the teachers for instruction, there should be other and different government. Every district should have its business board and its educational annex or adjunct, whether the latter be a number of persons or a single individual. The latter should have entire and untrammeled sway in perfecting and carrying out the methods of instruction in the schools. The former should be selected for business ability, honesty and zeal for the welfare of the public; the latter for scholarship, experience in teaching, and intellectual and social culture. Neither wing of such an organization will then be called upon to do more than that which it knows how to deal with, and we shall not have presented to us the oft recurring predicament which school boards get into, of wrangling for weeks over a set of text books of which the board knows little or nothing except what is injected into the minds of its members by the loquacious and plausible agent of a publishing company, or what one is pledged to approve by reason of promised gratuities of one or another kind made by the parties controlling a certain school-book. Nor would we so often witness that lamentable selection of instructors for the young which evokes the wonder and the chagrin of discriminating parents and patrons. The selection of text books should depend wholly upon their merits, and only they who by reason of familiarity with these, and a thorough understanding of the principles which underlie the topics taught, can determine for themselves what books are best for the schools; to such only should be entrusted this important matter. The public school system is far too conservative. The tendency of the age is toward specialism. We can most of us remember when the merchant was one who sold all manner of wares. The same store-keeper sold us hardware and dry goods, and crockery, and carpets, and drugs, and books, and confectionery, and shoes. Now we go to eight places for these, since it is demonstrated beyond peradventure that he who devotes his time and attention to one thing can better serve his patrons than he who devotes one-eighth of his time and attention thereto. One man designs our dwelling, another constructs its walls, another the roof, another completes the joiner's work, another plasters, another paints, another furnishes and another decorates it. He who does too many things does all clumsily. He who does one thing only usually excels in it.

This principle is recognized to a certain extent in our school system to-day, but not sufficiently. The state superintendent of schools is usually selected for his scholarly attainments and makes but an indifferent business manager. Now and then the State makes a mistake and puts into that position a man with a business capacity. As this official comes but seldom if ever in contact directly with the schools, his educational capabilities or deficiencies are not of special moment. The county superintendent, having little to do with the business administration of the schools, can and does give character to the instruction within his territory. He impresses the schools within his jurisdiction with whatever of excellence he may himself possess. Like the superintendent, like the schools. But his attention being extended over a wide territory, he can give personal supervision to none save at long intervals and during brief visits. The city superintendent of schools still nearer comes to our ideal of a superintendent of instruction. To conduct schools is one thing and to conduct instruction is another. The teacher is not to be relied upon wholly. He and she must have an authority to which they may go at any time, one near at hand and whose business it is to decide all matters pertaining to the methods of instruction used or discipline practised. As the giving of this matter into the hands of one man or woman alone is vesting the individual with too great power, as well as placing upon him too heavy a responsibility, it would seem that the most feasible, and proper, and successful plan would be to increase, if necessary, the number of our school controllers and then organize in each school district two distinct branches, one to control exclusively the business of the district and the other the teaching and moral government of those who attend as teachers and pupils. The province of each school board should be clearly defined, the one taking upon itself all matters purely of a business sort, the other all that pertains to the instruction and deportment, and in those matters where business and instruction are so blended that it would be difficult to say under whose province it fell, let the two act as a consolidated board. Thus, or by some similar method alone can we ever expect to have well balanced schools; that is, schools of which one can say that the government is secondary to the instruction, or vice versa, or that the educational status of the district has not been sacrificed to the business. A district may have fine and costly buildings and a most admirable system of school government and yet not be at all successful in turning out educated boys and girls. And again it may astonish the district annually with the intellectual attainments of its graduates, and yet thrust upon the community boys and girls so unevenly developed that they have no practical idea of the application of what they know to what they must come in contact with in busy life. We see schools of the one kind and the other every year. We find hordes of young men and woman graduated from our public schools every summer who are either so very practically developed that there is no room for the working of native genius, or who are so exactingly keen, intellectually, that the realities of life are continually jarring upon their sensibilities and making them continually miserable and well nigh useless, either to themselves or to those with whom they mingle.

It is not impossible nor impracticable to put into operation a system of school control which will give to one set of men the business of our schools and to another set the instruction of our youth. The two duties are as distinct as making canvass and putting a picture upon it. We choose lawyers, and doctors, and carpenters, and masons, and merchants, and even editors for our school controllers. Some of these may have a university education, and some may have taught a district school some time in their lives. But the majority of our directors could not conduct a class in grammar, or algebra, or chemistry, or even in geography or arithmetic. A large proportion of them could not correctly write a dictated sentence from the second or third readers used in the schools they are supposed to govern. What sort of judges of text books are these, and what do they know of the ability of a teacher to fill a position except from somebody's hearsay evidence? And yet such men are often the very best business men in our boards and are so valuable that we cannot afford to lose their business services. There is a twofold duty devolving upon school controllers, and the parts are so widely distinct that the same men cannot safely be entrusted with both. We need bankers in our school boards, because the best financial ability is demanded. We need builders, because the intelligent construction of school houses is very important. We need physicians, because the hygiene of a school should find intelligent looking after. We need lawyers to unravel the many legal tangles that school boards are getting into. We need mechanics of every grade, for there is much that is important in conducting schools which the non-mechanical eye would never detect. There is no class or condition of common sense man that will not come into good use in a school board. But we need something yet—ripe scholarship, wide experience in the instruction and training of youth, intellectual and social cultivation, familiarity with the principles and practice of teaching, a quick conception of fitness in an applicant for position in the schools, and ability to judge of the merits of a text book without getting his ideas at a literary pawnbroker's. Note how widely apart are the two sorts of duties which a school board is called upon to perform, and the reader must admit that our present system of giving all these divers sorts of work into the hands of the men we make school controllers of is all wrong. The only wonder is that we have such good schools in spite of duties so sadly misplaced.

